# A qualitative study into the perceived barriers of accessing healthcare among a vulnerable population involved with a community centre in Romania

**DOI:** 10.1186/s12939-018-0753-9

**Published:** 2018-04-03

**Authors:** Siân George, Katy Daniels, Evridiki Fioratou

**Affiliations:** 1 University of Dundee, School of Medicine, Dundee, Scotland, UK; 2 General Practitioner and Clinical Teacher, University of Dundee, School of Medicine, The Mackenzie Building, Kirsty Semple Way, Dundee, DD2 4BF UK; 3 University of Dundee, Ninewells Hospital & Medical School, Level 7, Room 003A (Lab Block, Corridor A), Mailbox 11, Dundee, DD1 9SY UK

**Keywords:** Healthcare, Access, Barriers, Vulnerable, Romania, HCAB, HABVP, Mixed-ethnic, Community Centre

## Abstract

**Background:**

Minority vulnerable communities, such as the European Roma, often face numerous barriers to accessing healthcare services, resulting in negative health outcomes. Both these barriers and outcomes have been reported extensively in the literature. However, reports on barriers faced by European non-Roma native communities are limited. The “Health Care Access Barriers” (HCAB) model identifies pertinent financial, structural and cognitive barriers that can be measured and potentially modified. The present study thus aims to explore the barriers to accessing healthcare for a vulnerable population of mixed ethnicity from a charity community centre in Romania, as perceived by the centre’s family users and staff members, and assess whether these reflect the barriers identified from the HCAB model.

**Methods:**

Eleven community members whose children attend the centre and seven staff members working at the centre participated in face-to-face semi-structured interviews, exploring personal experiences and views on accessing healthcare. The interviews were transcribed and analysed using an initial deductive and secondary inductive approach to identify HCAB themes and other emerging themes and subthemes.

**Results:**

Identified themes from both groups aligned with HCAB’s themes of financial, structural and cognitive barriers and emergent subthemes important to the specific population were identified. Specifically, financial barriers related mostly to health insurance and bribery issues, structural barriers related mostly to service availability and accessibility, and cognitive barriers related mostly to healthcare professionals’ attitudes and discrimination and the vulnerable population’s lack of education and health literacy. A unique theme of psychological barriers emerged from both groups with associated subthemes of mistrust, hopelessness, fear and anxiety of this vulnerable population.

**Conclusion:**

The current study highlights healthcare access barriers to a vulnerable non-Roma native population involved with a charity community centre in Romania. The “Healthcare Access Barriers for Vulnerable Populations” (HABVP) model is proposed as an adaption to the existing HCAB model to account for the unique perceived barriers to healthcare for this population. Recommendations for future resolution of these identified barriers are proposed.

**Electronic supplementary material:**

The online version of this article (10.1186/s12939-018-0753-9) contains supplementary material, which is available to authorized users.

## Background

Vulnerable populations worldwide are subject to poorer health outcomes [1]. Dimensions of vulnerability are vast so defining a ‘vulnerable population’ is difficult. Factors that can lead to vulnerability include: socioeconomic status; geography; gender; age; disability; health status; ethnicity; culture; vulnerable to abuse; vulnerable to human trafficking (sex, slavery, forced labour); or are in adverse circumstances unable to cope with and recover from the impact of a disaster [2]. Such populations experience numerous barriers to accessing healthcare [1]. In this study, barriers to healthcare access will be explored in the context of native vulnerable European populations, encompassing both Roma and non-Roma communities.

The Roma population comprise Europe’s largest ethnic minority and are considered a vulnerable population throughout the world. Within Romania, the Roma community make up approximately 3.1% of the population, however figures are usually underestimated so this may be as high as 11% [3]. The non-Roma community studied here were ethnically Romanian.

Some of the healthcare access barriers identified within the literature correspond to aspects of published healthcare access models. Carrillo et al. proposed the ‘Health Care Access Barriers Model’ (HCAB) [4] which targets three types of measurable and modifiable barriers: financial, cognitive and structural. Within these, financial barriers included health insurance, whilst cognitive barriers included topics such as knowledge, communication, language and health literacy, and structural barriers included issues such as waiting times, availability and transport. The HCAB model was originally designed for administrative purposes, but has the potential for adaptation for use by health and social care staff. Outwith the HCAB model, additional barriers may include cultural and attitude issues.

Economic factors play a major role within private and health insurance based systems. Personal financial funds are needed to access both private care and health insurance. Lack of health insurance can have a significant negative impact on access to care in many countries [5, 6]. If insurance has been paid, the Romanian system can offer some benefits of universal healthcare coverage. However, despite having insurance, additional costs are often present when accessing care, leaving those in lower socioeconomic positions unable to afford services. In addition to the initial cost of health insurance and subsequent medicine costs, expected bribes or off-the-record payments create a further financial barrier [7]. Consideration must also be given to what is included in a health insurance policy as some services, such as mental health services, are often excluded from Romanian policies [8]. There is a distinct similarity between the poorest population quintile and the Roma population regarding ability to afford healthcare [9]. This may be due to the poorest quintile being more likely to receive social assistance. As well as this, further implications of socioeconomic status is echoed by Masseria et al. [10].

Structural barriers encompass the healthcare system’s availability for both external and internal factors to the immediate healthcare facility. Some of these barriers may include: transportation, geographical location, system organisational barriers, general availability of services, health information, waiting times and health infrastructure. Structural barriers can occur independently or can overlap with other major barriers, such as, economic barriers.

Vulnerable populations often live in isolated communities which causes difficulty travelling to the desired healthcare service location due to transport costs or lack of transport links and missing appointments due to transport delays [11]. Alongside geographical difficulties, the availability of information regarding healthcare entitlements, availability or resources within Romania’s isolated or vulnerable communities is unknown, as no such study has been published. Campaigns to educate vulnerable communities on their healthcare rights is needed to move forward with improving care access and equality [9]. Furthermore, the World Health Organisation (WHO) recognises that structural barriers and poorer health infrastructure within health systems need to improve to ensure health equality for those vulnerable to poverty and social exclusion [12]. Structural barriers cause significant problems with health screening programmes; specifically, cervical cancer screening programmes, where targeted female populations in both Romania and Bulgaria are unable to access the services in the first instance due to the system’s design. Furthermore, women experience long waiting lines and mis-communications with staff who often refuse to carry out procedures unless the person was deemed “sick” [13]. Communities living within lower socioeconomic areas, from smaller settlements or an ethic minority were less likely to attend screening and more likely to have perceived costs with the procedure. These costs include financial costs and cognitive fear of the test due to a lack of available information for these groups provided by national health programmes and by individual healthcare professionals. Additionally, other national cancer screening programmes such as colorectal and breast have not been totally implemented within Romania [14]. Romania also lacks legal frameworks and regulatory bodies impacting the ability to promote efficient and quality care [13].

Communication and language barriers are highly prevalent because of the evolution of diverse populations due to migration [12, 15]. Challenges exist to ensure equity and awareness of these groups whist not forgetting native impoverished populations [16]. Language interpreters are key to minimising these barriers without which, difficult situations for both patient and professional arise and impede the access and delivery of healthcare [16]. Trust in the interpreter is imperative so finding the right person is crucial [16]. Other communication problems may arise due to differing levels of education and healthcare professionals lack of understanding about poverty, patient situations and associated multifaceted needs [15]. Poor maternal education accompanied by poverty  has been shown to be a negative determinant of birth and health outcomes in Roma mothers when compared with non-Roma [7, 17].

Cultural and attitude barriers mostly incorporate aspects of discrimination and include dimensions of: socioeconomic position; individual beliefs; social groups; language; race; ethnicity; and religion. EU law protects against discrimination on the grounds of sex, racial or ethnic origin [18], but nevertheless, discrimination received from healthcare professionals extends further than just socioeconomic and educational factors [19]. Cultural or ethnic discrimination is widespread and linked to poorer quality of care [7, 20]. Discrimination through stereotyping is also prevalent with Roma being judged by healthcare staff based on their education and unemployment rates causing mistrust toward professionals [6, 10, 21]. Negative experiences from mistreatment or prejudice result in reluctance to attend services and negatively impacts perception of health [5, 21, 22]. Cultural barriers also have a strong relationship with community willingness to access appropriate care [23]. Policy-makers regard the Roma to misunderstand how the system works, and to misinterpret discrimination as the reason for feeling victimised [7]. However, staff education surrounding cultural competency could increase uptake of desired services by culturally diverse populations [22].

In conclusion, minority vulnerable populations throughout Europe and further afield face various barriers accessing healthcare. This study is undertaken within East Romania due to its known impoverished areas alongside the country’s on-going healthcare crisis [8, 18, 19]. Furthermore, the gap between different socioeconomic groups with respect to the level of their unmet medical needs between Romania and the EU is unequally distributed; Romanian vulnerable groups are confronted with difficulties beyond those faced by their counterparts elsewhere [24]. Negative health outcomes have been portrayed and documented as a consequence of numerous barriers for European Roma communities and vulnerable communities living outside of their native country [11]. However, the potential healthcare barriers faced and health outcomes of different ethnic vulnerable European or Romanian non-Roma indigenous communities has yet to be as significantly acknowledged. Therefore, following the HCAB model [4], this study aims to detect and explore three things: firstly, the perceived barriers to accessing healthcare for a vulnerable population of mixed ethnicity in Romania, secondly, assess how these barriers may impact health according to this community, and thirdly, make recommendations as to how some barriers may be overcome.

## Methods

### Design

A qualitative research methodology was used as the study aimed to be exploratory in nature, detecting and examining personal experiences to identify detailed themes and opinions from participants. Semi-structured interviews were chosen to allow an in-depth exploration of their personal experiences, attitudes and views can be gained. It also provides an appropriate format for discussing sensitive subjects whilst allowing a degree of flexibility to change questions to address areas important to each participant [25]. Furthermore, as a large percentage of the targeted participant cohort are illiterate, methods involving reading or writing were inappropriate. Focus groups were also unsuitable due to the desire to ensure that quieter participants have their voice heard.

Two similar question guides [see Additional file 1] were created to address the project aims for each cohort interviewed. The two cohorts selected included family members whose children attend the centre and staff members who work at the centre. Open-ended questions to explore themes were the main-stem of the interview guides but the HCAB model themes were assessed for by the use of focussed questions. Question guides were subsequently discussed with the other authors *[K.D. and E.F.]* to assess their appropriateness to meet study objectives.

Ethical approval was granted by Dundee’s University Research Ethics Committee (UREC).

### Sampling

The study population were adults living within or working with a vulnerable community residing in an area of East Romania. Convenience sampling was used, with the targeted population being reached through a charity organisation who run a children’s community centre in the area. This method was chosen because of existing relationships with members of the target population, a community where there is a high degree of mistrust of ‘outsiders’. The high degree of trust between the target community and the centre granted easier access to this community.

The children’s community centre aims to keep children from vulnerable families in school through providing a safe place for them to do school work and play as well as providing a meal; showers; educational support; life skills and help with clothing, but does not provide healthcare. Access to the centre was established through a co-author’s *[K.D.]* previous volunteer work.

As previously mentioned, two target cohorts were used:

1. Family members. This cohort included mothers or fathers over the age of 18 of children attending the centre.
2. Staff members working at the centre, who are considered key informants of the community because they have been working among this community for over 15 years.

### Recruitment

Family member recruitment began at a parent meeting at the centre. The study was presented and participant information sheets read aloud in Romanian. Confidentiality of the interviews and voluntary involvement were emphasised. If family members wanted to participate, a choice of translator was given, either a staff member from the centre or an independent translator, to ensure comfort and openness amongst participants.

Fifteen families were represented at the parent meeting, of which, fourteen agreed to participate. An additional ten families of children attending the centre who did not attend the parent meeting were later invited to participate on house visits or by telephone by the centre’s social worker and the researcher. Here, the same protocol was followed. All ten agreed to participate. All of the family members chose a staff member as their translator.

Thirteen staff members were informed of the study during a staff meeting and individual participant information sheets were provided in the language of their preference (English or Romanian). Over the subsequent 4 weeks, participating staff interviews, with a translator of their choice, if they wished to have the interview in Romanian, were conducted. All participants were advised that no reward or consequence for participation or non-participation would ensue.

### Data collection

Data was collected through face to face semi-structured interviews that took place with individual participants, the researcher and a translator (if needed) in a private room at the community centre in January and February 2016. This setting was a safe and familiar environment to encourage conversation with participants. The interview process for family and staff members was the same. Commencing every interview, the participant information sheet was read, confidentiality was explained, consent for audio recording with a dictaphone was obtained and consent forms were signed. Interviews were saved to an encrypted USB device.

### Sample size

Staff and family cohort numbers required to reach data saturation, according to Guest [26], were estimated. The number of interviews estimated to reach data saturation in terms of no new information or emerging themes [26] was 10–15 family members, and 5–10 staff members.

Following the recruitment process, 24 family interviews were scheduled to allow for cancellations or non-attendance based on advice from the centre and indeed a number of participants did not attend. Reasons for non-attendance included illness, conflicting schedule or forgetting. When it was considered that the point of data saturation was reached, 11 family members and 7 staff members had been interviewed which included two pilot interviews. Subsequent to these numbers no further interviews were held.

### Piloting

One staff and one family pilot interview were conducted within the targeted research group prior to subsequent interviews to establish any weaknesses within the interview design and if the information gathered would answer the objectives. Consequently interview questions were amended to remove ambiguous or complicated words due to low levels of literacy and education within the family member group. Pilot interviews were deemed successful and so were included into the results.

### Role of researcher

Qualitative research may exhibit an element of researcher bias due to the nature of the analysis and the interaction the researcher has with the participants at the interview stage. Therefore, researcher reflexivity was used continuously alongside self-scrutiny and considered across all stages of the project in an attempt to minimise bias and allow improvements for future interviews.

### Coding and thematic analysis

Thematic analysis was undertaken using the six phase steps described by Braun and Clarke [27].

Deductive thematic analysis was used at first pass to identify HCAB model themes of financial, cognitive and structural barriers. Transcripts were then analysed with an inductive approach to identify new emerging codes and themes not previously identified within the HCAB model. The HCAB model was flexibly used with the potential for adaptation to demonstrate this study’s findings within the results write-up. To ensure reliability of the data, two steps were taken. Firstly, triangulation of sources was used; by interviewing both family and staff members of the area. Secondly, a different analyst independently reviewed and analysed the data to cross-check it with the findings of the researcher, however consensus on final theme categorisation was reached as a team. Quotes for illumination of context and or meaning as described in results and discussion section of the paper were chosen at random from within their selected themes and subthemes.

## Results

### Participants

By the point of data saturation, eighteen interviews were completed. Eleven family member interviews and seven staff interviews. All family participants and six out of seven staff participants originated from Romania. The remaining staff member originated from the USA but has been living in Romania since 1999. Of the family interviews conducted, seven out of eleven participants were ethnically Roma, and four Romanian. All staff members interviewed were non-Roma. Staff members held differing professions and positions within the centre. These included: teachers, an educator, social worker, regional coordinator, janitor and centre coordinator. For confidentiality purposes, quotes are anonymised. Family interviews are labelled F1-F11 and staff interviews are labelled S1-S7.

### Interview results

Table 1 highlights the themes and subthemes found within the data following both deductive thematic analysis according to the HCAB model, and additional inductive thematic analysis.

**Table 1:** Themes and subthemes within the data

THEME	SUBTHEMES
Financial Barriers	1. Health Insurance 2. Employment Status 3. Affordability 4. Bribery 5. Competing Priorities
Structural Barriers	1. Service Availability 2. Accessibility 3. Waiting Times 4. Adults versus Children
Cognitive Barriers	1. Healthcare Professional Attitude and Mentality 2. Language and Communication 3. Vulnerable Population Discrimination 4. Cultural Discrimination 5. Health Literacy
Psychological Barriers	1. Mistrust 2. Hopelessness 3. Fear 4. Anxiety

### Financial barriers

Multifactorial components of this barrier exist and are a major determinant for ability to access healthcare. Health insurance *(subtheme 1)* is a crucial barrier; without insurance, primary care cannot be accessed therefore, secondary care referrals cannot be made, and only emergency care can be sought.

*F5: “because she doesn’t pay the, insurance, she doesn’t have any insurance, she doesn’t have family doctor”*

Health insurance is not required to access private care, however payment is necessary which is mostly unaffordable to vulnerable families. Some of the family participants state that they managed to overcome having no insurance by creating a relationship with a family doctor who agreed to consult with them regardless of insurance status. However, they accepted that this relationship did not guarantee any other healthcare access points indicative of having insurance, such as secondary care referrals or subsidised prescriptions.

*F10: “My family doctor sent me to the hospital to be checked in 3 times, but at the hospital they say I don’t have insurance so they cannot accept me”*

Employment status *(subtheme 2)* was shown to play a large role as unemployment is rife within the community and impact upon the ability to acquire health insurance. All participants indicated that they have had issues with the ability to pay for either services or medicine – regardless of ability to access a doctor.

*S2: “and it's another aspect for these families, some of them they don't have a job, and they don't pay their medical insurance, so they can't have a medical doctor, and they can’t afford to pay”*

Affordability *(subtheme 3)* extends to investigations and type of service desired. Dental care was commonly needed, but dental services are not covered within health insurance policies and costs are too high for most. Bribery *(subtheme 4)* and corruption within the healthcare sector was described by all but one interviewee. This did not solely involve the financial implications of paying bribes, but also impacted the standard of treatment and attitude received from healthcare professionals. Participants acknowledged that for a community who already cannot afford healthcare, additional bribery payments add to their problems and emotional stress.

*F4: “she knows that God says not to give bribes, but if you don't give bribes in the hospital they will let your kids die (…) i'd have to sit in the hallway until I have paid them something, they wouldn’t let me in until I have paid them something.”*

With limited income, competing priorities *(subtheme 5)* of money controls whether participants access the necessary care. Both cohorts explain that prioritisation extends to every day life and community members prioritise commodities like food or heating, or choose to invest in their children - rather than look after their own health.

*S2: “for example, we had a project and for those who participated, like 80% from the, the parent’s meeting, they received some money, and I asked one of these ladies, if she puts some money, just for herself to, in order to have a medical check-up and she said oh no, I just I bought food, for heating, for heat, yeah and I bought some clothes for my kids and I’ll pray to god to help, to have mercy on me”*

### Structural barriers

A general response amongst participants was a belief that healthcare services were plentiful, but not all were accessible, despite health insurance status, as many are provided by the private sector. Nevertheless, those with insurance face fewer problems. However, some participants, specifically shown by S3, believe the healthcare services available in their city are not substantial for the needs of the community.

*S3: “the system is, is still antiquated, and under developed for the needs that they have.”*

Staff discussed how quality care can be accessed if one has more financial resources. Although this was in contrast to most participants who believed that service availability *(subtheme 1)* within the city was substantial, but the services are of a poor quality. It was found, as described by S1, that when a serious health issue occurred, they tried or wished to access care in an alternative city as the perceived quality of services was higher, showing an inequality of access depending on the location of a person’s inhabitancy.

*S1: “if you want something good, better than [named own city], you go to other cities like [named city] or [named city]. So if you have major surgery, and you are very frightened you go to see like we say, a better hospital or better doctor, and [named city] or [named city], we do that.”*

Service availability is thought by the community to be influenced by the prevalent staff shortages and salary disputes within the healthcare sector across the country.

*F7: “The doctors are, are not paid enough to do their job, their salary is quite low, low, that's why they are not interested in being more responsible and being more involved and doing their best”.*

*S2: “We have specialists, like good doctors, but in [named own city] yeah, err, now the unemployment percent it is really high and income is really low and they prefer to go outside, so, the corruption is getting bigger.”*

Both cohorts also explain how a service may not be available in this part of Romania and the only option is to travel elsewhere. However, travelling to another city is often not feasible because of waiting lists and the costs involved. Within the community area itself, participants described the multiple options of transport (buses, cabs, maxi taxis) and the ease of travelling to services. Consequently, participants believe that geographically, there is good accessibility *(subtheme 2)* of services within the city. However, accessibility of care as an adult versus a child *(subtheme 4)* was considered more difficult, as children up to the age of 18 in Romania are theoretically entitled to free healthcare.

*F5: “So the kids have a medical insurance because they have to till they have 18 years old, so they go to the same doctor, but she doesn’t have medical insurance so that's why they don't pay attention to her, the medical, the doctor, doesn’t pay attention to her. But they, he has to pay attention to the kids.”*

Nevertheless, children’s treatment or medicine plus services out-with health insurance such as dentists, private care and opticians still needs payment, causing similar accessibility problems experienced by adults. Furthermore, barriers of queues or waiting times *(subtheme 3)* were considered to affect adults and children equally. Within this community, if a participant is placed on a waiting list to see a specialist or receive treatment this was perceived as a positive step in accessing care. However, queues within hospitals, especially for those solely eligible to seek emergency care, pose a hurdle.

*F3: “So they gave me just one pill to calm down, after this I left, because I had to waited for hours and hours and hours there, it is very crowded in there.”*

S4 describes the controversy with the queuing system with people admitted ahead of others purely because they know the doctor. This contributes to the ideology the community hold about Romania’s services, doctors and the Romanian system in general.

### Cognitive barriers

Fear of negative healthcare professional attitudes and mentality *(subtheme 1)* due to previous bad experiences was shown to cause avoidance of care or premature departure from hospitals without receiving adequate help. Interview accounts from both cohorts portray doctors ignoring, shouting and speaking derogatorily towards patients.

*F3: “many times I went to emergency, and there was times I got mad and just leave the emergency because nobody pay attention to me.”*

These attitudes were seen to alternate depending on situational circumstances. As such, the attitude and judgement received from healthcare professionals showed to correspond to the vulnerability status of those in the community for example, being poor, uneducated or impoverished and thus transpired as vulnerable population discrimination *(subtheme 3).*

*S5: “there is also issues in this part of town, where doctors treat this group of people differently, or they view them differently, because of poverty, because they have low education, because they have no money and that influences things as well”*

Despite this, staff accounts of mistreatment from health professionals were also uncovered, as shown by S1. Other accounts state that doctors believe to view themselves as superior and to not treat patients humanely.

*S1: “the people don't speak nice with them and that's a problem for them. Because when they have a health problem, the first thing they think is not to go to a doctor because they are frightened, because they are scared they will not talk with them or something like that”*

Language and communication barriers *(subtheme 2)* involved the use of medical jargon and language that the patient is unable to understand, negatively impacting the doctor-patient relationship and resulting in the consultation being misunderstood. Negative discrimination was shown to extend further than just socioeconomic factors. There is a large percentage of ethnically Roma population within the area, and many participants described cultural discrimination *(subtheme 4)* based on the stigma attached to this ethnicity.

*S3: “if they are Roma background there is even more resistance to them, there you know, racism in the culture in general, because they are poor they are going to be err, more marginalised because of lack of education, more marginalised, and there is already a mentality of if you're the doctor then you are superior”*

It is suggested by F10 that Roma can be treated better if they have money, linking to healthcare professional attitude *(subtheme 1)* and how it changes depending on situational circumstances.

*F10: “There are many doctors, who, if they see me, like a Gypsy, will not respect me or even insult me. This is for Gypsies who are really poor, there are some Gypsy who have money and they will get treated well.”*

It is shown here that low levels of education increase the risk of discrimination, and can lead to lower levels of health literacy *(subtheme 5)*, which spans a number of issues. This was demonstrated through unfamiliarity with entitlements or rights as citizens and unawareness of how the health system works and how to navigate it. The consequences of general illiteracy was described as an inability to read health information leaflets, posters or similar materials often displayed in healthcare establishments as well as instructions on prescriptions. Despite a number of the family members being illiterate, they did not identify this barrier, only staff members highlighted it. One family participant did highlight how her lack of education left her unable to dial the emergency services for an ambulance whilst in labour.

*S3: “So not knowing how the system works, that would be 1, and then 2, not knowing if they’re given a diagnosis, not knowing how to interpret that”*

However, some family participants knew their entitlements and how the health system worked. Others were unsure of their entitlements because they had no interest in knowing, not because they were unable to find the information.

*F10: “I’m not very sure. I didn’t ask, I wasn’t curious.”*

Decreased awareness of prevention facts, lack of basic health and lifestyle information, unawareness of long term consequences and not valuing health or recognising health importance were also found, alongside, the loss of the ability to distinguish cause and effect of health issues. This, again, like other cognitive barriers, was solely acknowledged by staff members.

*S5: “So, I feel that they don’t necessarily think about what they are doing now and the impact that is going to have on the longer term. And so it's actually, a sort of mentality, sort of way of life, that actually they also then gets passed onto the children. So there's people who will cough and they'll be unwell and they'll continue to smoke and so they smoke in the same room as children. But also education, also about things like how to keep their house clean, so hand hygiene, personal hygiene, and these kind of issues as well and the impact that that has.”*

### Psychological barriers

In addition to the three themes (financial, structural and cognitive) described within the HCAB model; a further theme of psychological barriers emerged during inductive thematic analysis. Four main subthemes within this included: mistrust, hopelessness, fear and anxiety. These added another dimension of an emotional impact to the community’s problems.

Both negative and positive personal experiences shaped the views and mentality community members have of the city and its healthcare services. The attitudes of healthcare staff have led many participants to believe all healthcare practitioners are disrespectful. As such, they are shocked if they are treated otherwise and defensive mechanisms are prominent when interacting with healthcare professionals. This mentality causes mistrust *(subtheme 1)* and impairs creating a positive doctor-patient relationship. Mistrust and lack of faith also emerged about the Romanian healthcare system, its doctor’s and diagnoses. Participants described misdiagnosis, discrepancy between diagnoses depending who they asked and the same diagnosis constantly given for different people.

*S7: “in many many cases, we discover that they have this belligerent attitude (...) just because they have been treated badly they have had bad experiences, but we try to teach them do not assume that everyone, because not true, there are people that treat them like human beings, so we try to work on that, but it's hard because when you have I don’t know, 80, 90% bad experiences, it is hard to believe that”*

Furthermore, community members were shown to have a preconceived negative view of healthcare services and staff in this area of Romania, discouraging family members from accessing care believing only poor care is available. Hopelessness *(subtheme 2)* for gaining help was seen as family members are aware they need help but have no opportunity to, for example, if they are uninsured. This hopelessness extended to the healthcare system; people hope for a change in the system, but are not optimistic about any reform.

*F3: “Yes, I would like to, but I have no chance. (…) I don’t feel okay, I don’t feel good. I don’t think I have a chance,*

Added to this, some family members are frightened or anxious about seeking a diagnosis because they fear *(subtheme 3)* they have no chance of gaining access to a doctor or subsequent treatment needed. Fear of being diagnosed with a life changing illness also leads to a failure to seek healthcare, because what follows the diagnoses is deemed to be outwith their control.

*F10: “and I am afraid they will discover some other issues, like, maybe I have cancer, maybe I have diabetes which I suspect I might have and I don't want to find out. (...) I would rather like die suddenly and not know”*

Finally, complexity in the operation of, and corruption within, the healthcare system created psychological barriers of stress and anxiety *(subtheme 4)*. Bribery was an issue for both family and staff and so is a barrier that disregards vulnerability. Anxiety was also induced due to the current staffing shortages and the impact that had on available specialists and care.

*S6: “I don’t know what to do exactly, I don’t know how to, just because I don't want to be treated badly, I want to bribe them, bribe the doctor, but I don’t know exactly what to do, and that's my dilemma and my anxiety.”*

### Barrier solutions

Both family and staff members explained solutions and resolutions sought by the community in order for them to avoid accessing care. For example, some family members use self-help or herbal remedies.

*F3: “and if he has an ongoing cough, I use the example, I give him onion tea.”*

Alternative coping strategies used by the family participants and aided by the community centre are highlighted below within the main themes.

#### Financial

*F6: “I am borrowing money.”*

*S4: “And then, errm, the other thing, they can take small loans and they come now and then for medicine, to take a small loan for medicine, like, and they will pay it back”*

#### Structural

*S3: “I’II try to find doctors I know or know people I know and get them to talk to them”*

*F6: “in this situation, she reached to (staff member) for milk and for the baby, and (staff member) help, help her”*

#### Cognitive

*S2: “Oh yeah, sometimes they come here and ask us for some advice”*

These solutions show the community members utilise the many services provided by the community centre, some of which include education for children, counselling services, advice, information read or small loans of money. Undoubtedly, without the community centre this vulnerable population would struggle further, and although the centre is able to help with some barriers, S7 discusses the difficulty of changing major cognitive barriers such as mentality and discrimination.

*S7:“I mean to provide something for people is not very hard, but to change mentality (...), it’s a life time process.”*

Further to these solutions, psychological barrier solutions need to be accounted for. There were no solutions for these volunteered during interviews. Alternative barrier solutions such as those for financial, cognitive and structural issues can all help with the psychological aspects of community member’s disengagement with healthcare, however, similar to the ability to alter mentality, fundamental personal emotions can take decades to change.

## Discussion

### Summary

This study aimed to bridge the gap and identify healthcare access barriers for both ethnically Roma and ethnically Romanian groups within a city in Eastern Romania. To do this, a model recognising modifiable healthcare access barriers for vulnerable populations was utilised [4].

**Fig. 1:**
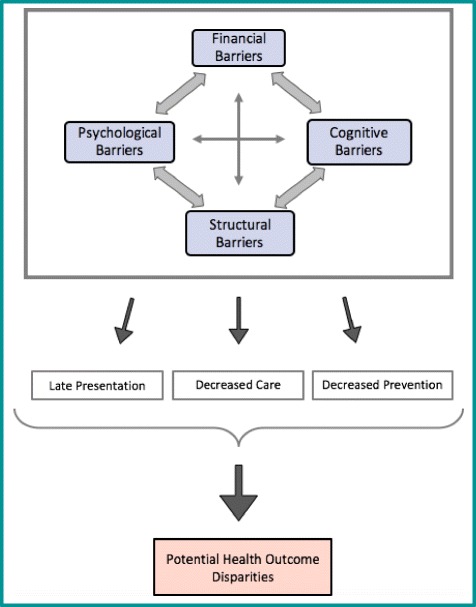
The Healthcare Access Barriers for Vulnerable Population (HABVP) Model. This is a three tiered model showing the connection between findings with directional arrows. It demonstrates: a top tier of intertwining barriers found to impede healthcare access (financial, cognitive, structural and psychological); a middle tier highlighting the three main consequences to the barriers (late presentation, decreased care and decreased prevention); and a bottom tier depicting the overall outcome of the previous factors (potential health outcome disparities)

Four main barriers were found impeding healthcare access. The financial, cognitive and structural barriers found are consistent with the HCAB model used [4]. However, the additional finding of psychological barriers is new. As such, a new framework has been created for this study’s findings, shown in Fig. 1*,* and is named the ‘Healthcare Access Barriers for Vulnerable Populations Model’ (HABVP). Most studies fail to mention or acknowledge the poorest or most vulnerable within society out-with ethnicity, who too are subject to health outcome disparities on-par or worse off than the Roma community. The current study acknowledged a mixed ethnic population and added the findings of psychological barriers to healthcare access to the literature, alongside other dimensions of financial, structural and cognitive barriers.

#### Study strengths and limitations

Naturally, this research is based on people’s perceptions of health and well-being, whilst this was the intention of the study, health outcomes for any condition have not been measured, therefore cannot be supported with original quantitative data, however, clear evidence exists elsewhere that healthcare access barriers for vulnerable groups leads to poorer health outcomes [1, 28]. A study strength was that family participants were either from a non-Roma or Roma background, however all staff participants were non-Roma in origin. Despite staff members being highly aware of the issues faced by the vulnerable community, having a staff member of Roma origin may have added a different perspective to the staff interviews.

Although one of the authors had previous links with the centre, this was through volunteer work and not a working contract. Furthermore, all interviews were conducted by a member of the research team who had no previous relation to the centre or the participants, both of these circumstances limit bias and adds strength to the findings. Additionally, piloting interviews by the same researcher within the targeted population allowed the researcher to gauge if the questioning was understood and appropriate from the outset.

The identified HCAB model added further value to this study as it led to the creation of the more comprehensive HABVP model. Two further models were researched and acknowledged but not incorporated into the study. Andersen’s ‘Behavioural Model of Health Services Use’ [29], integrates non-modifiable barriers which are not assessed in this study and include, demographic, environmental, social structure and behavioural factors and secondly, Devoe et al. [30] demonstrate a typology of barriers but solely incorporate economic factors, whereas this study aimed to explore a multitude of barriers.

#### Discussion of study findings

Based on this study’s findings, financial barriers were undoubtedly the commonest barrier hindering care access described across both cohort groups. The community were frequently able to consult with a doctor but subsequently unable to afford the medication prescribed, which is consistent with UNICEF’s and Idzerda et al.’s findings [9, 30]. UNICEF however did not differentiate the vulnerable non-Roma in society from the general non-Roma population, like this study did.

High unemployment rates cause a cascade of effects with lower household income and importantly prevents access to health insurance because this is automatically paid for from a person’s salary. Members of the community in this study often turn to illegal work to earn money, meaning some care may be bought, but ultimately health insurance is pivotal at determining the entry and volume of care received, regardless of ethnicity. In contrast to these findings, Masseria et al. and Kühlbrandt et al. found that the Roma population within Central and Eastern Europe are significantly more likely to lack health insurance compared to non-Roma living in a similar area [10, 21].

The impact of bribery within the Romanian healthcare system is entwined throughout this study’s findings, and impacts both patient and professional. Healthcare professionals were deemed to base the quality of their communication and care upon the ability of a patient to pay a bribe and their socioeconomic status. Low wages within Romania and the desire for better wages, working conditions and more opportunities elsewhere [31] helps to drive the ongoing bribery payments made, and citizens continue to hold the mentality that they pay for the quality of care they receive, regardless of the reality.

Attitudes of healthcare staff are another significant barrier in this study alongside other vulnerable groups which hinders access to care and increases discrimination [19]. Discrimination based upon education or poverty for indigenous populations as found here, is not recognised across Europe. Some results suggest patient background may be irrelevant within Romania but experiences of frequent discrimination result in delayed or avoidance of seeking medical treatment, leading to worse mental and physical health. Conversely, some data gathered showed few doctors to have positive attitudes towards vulnerable community members, namely, family doctors offering services for free due to their personal kindness. Using the new HABVP model, healthcare professionals can be aware of the impacts of their own practice of discrimination and make a conscious effort to modify their behaviours.

Despite qualitative research focusing on personal perceptions and experience, psychological factors and personal emotions are not described elsewhere as barriers though were identified as such within this research. Negative emotions from previous experiences act as the stimulant for future healthcare disengagement, affecting overall health. Due to a lack of data, it is unknown if the negative perception of the city’s public healthcare system by the community and staff is justified. People do not believe the system can change so they do not modify their mentality, despite interactions with services. It is unknown whether this is a healthcare specific mentality or more general, but a community mentality is passed through generations.

Lack of education or health literacy spans across a range of topics including awareness of how the health system works, entitlements and understanding preventative health measures. The least educated in Romania, are approximately five to eight times more likely to be at risk of poverty or social exclusion [28], which increases the risk of discrimination within the health system. Information regarding healthcare entitlements, availability or resources are required to facilitate access to healthcare and isolated vulnerable communities do not always receive this, as found elsewhere [6, 7].

Conflicting opinions were found regarding service availability and quality in the area.

But regardless of this, inaccessibility remains high. Other services namely, pharmacy access was not an issue for the community, but the cost of medication causes problems. Dental care emerged from the data as a highly needed service, and like other European countries, avoidance of dental services for financial reasons is the most important contributing factor to poorer dental health [32]. Furthermore, the disadvantaged and marginalised populations carry the greatest burden of oral disease, which has strong associations with non-communicable chronic diseases and impacts long term health and quality of life [33].

The HABVP depicts three main consequences of the documented barriers: late presentation, decreased prevention and decreased care. Although these were not directly assessed, these were eluded to by participants, and could explain why Roma within Romania are recorded to have higher mortality rates and lower life expectancy from birth alongside other adverse health outcomes when compared to the majority of the population [24, 34, 35]. The only additional barrier faced here by Roma was cultural discrimination. Cultural discrimination from healthcare professionals must be accounted for under cognitive barriers due to its powerful impact on healthcare access. However, no additional barriers were found for non-Roma.

This study identifies that education could play a pivotal role in both improving healthcare access and reducing the risk of unfavourable health outcomes. Therefore, recommendations for barrier resolution and potential consequent improvement in health outcomes, using educational methods have been proposed and include:

1. Community level.

Firstly, provide basic health and lifestyle education from a healthcare professional with approximately three, one-two hour sessions, for all willing community members. This may involve: dispelling common health myths; personal hygiene teaching; importance of preventative measures and early presentation; basic first aid training; key lifestyle factors; long term health consequences; and citizen care entitlements.

Secondly, educate families about bribery within the system and what they are supposed to pay for and what they are not.

2. Healthcare staff level.

Firstly, educate healthcare staff about what is appropriate behaviour regarding bribes. It is of note that a higher level change and intervention is needed with this resolution. However, currently, bribery is illegal but the law is not enforced. The doctor-patient relationship is crucial to effective healthcare service, but this is compromised within the bribery system.

Secondly, discrimination and negative attitudes received by non-empathetic healthcare professionals must be addressed. These professionals should obtain training in two areas. 1) Cultural competency surrounding the Roma population, and 2) staff development about the impact discrimination has on the care-giving process and how negative experiences have future unfavourable connotations. Staff at the community centre could be in a position to deliver this education to healthcare staff due to the trust, relationships and knowledge they have of the community.

#### Research implications and future research

The new HABVP model depicted in this study may not exhaustively identify all aspects of health disengagement and might not be applicable to every vulnerable population as it does not depict the connection between numerous other unmodifiable healthcare determinants of disparities and health outcomes. Therefore, further research is needed to address other non-modifiable healthcare determinants of disparities, such as: genetics, gender, individual behaviour and physical environment; and the impact these have on healthcare access and overall well-being. The HABVP model does nevertheless show the effect that barriers, either singular or multiple, can have on accessing healthcare. This is the first time the model has been applied within Europe, and so has the ability of modification for each population and can facilitate the design of community health interventions, aiding barrier resolution.

## Conclusion

The current study opened a unique door into the lives of the specified ethnically diverse vulnerable community and explored their perceptions of barriers to accessing healthcare which allowed us to appropriately address the research aims.

The HABVP model and this study increases the understanding of the root causes for vulnerable populations disengagement with healthcare and enables professionals to be more proactive and holistically consider the needs of the population they serve. Access-related problems need to be continually focused upon by both healthcare professionals and policy-makers to decrease the prevalence of healthcare avoidance by those most in need. However, this study also identified needs for those people looking after the vulnerable population within the community centre. Both cohorts must be considered as there is a need to care for everyone in the system.

Interview findings suggest that an increase in education could be a key route to barrier resolution in the vulnerable community. Basic education and teaching could reap a host of benefits including: greater knowledge of basic health and lifestyle factors; knowledge about how to navigate the healthcare system; engagement with health services, and; lead to increased employability prospects with financial opportunities. As the centre has such strong relationships with the community, their provision of education could be a sustainable solution for the community.

Finally, this paper provides insight into the number of problems the most vulnerable from different ethnic backgrounds within one community face when accessing healthcare. With the inequality gap within society increasing, improving access to quality healthcare is essential for making steps towards health equality. The community centre provided a good source of data, and in return, the findings of this study are relevant to help aid the community centre develop their work locally. Furthermore, this research helps provide steps needed to progress towards the end goal of health equality, and it can be used as a guide for vulnerable communities internationally to identify areas of healthcare access barriers and help facilitate community health interventions for barrier resolutions.

## Additional file

Additional file 1: (1) Interview guide for family members, and (2) Interview guide for staff members. (DOCX 103 kb)

## Abbreviations

HABVP Model: Healthcare Access Barriers for Vulnerable Population Model
HCAB Model: Health Care Access Barriers Model
