# Supplementary material for: A qualitative study into the perceived barriers of accessing healthcare among a vulnerable population involved with a community centre in Romania
Source: Int J Equity Health. 2018 Apr 3;17:41. doi: 10.1186/s12939-018-0753-9 (PMC5883264; doi:10.1186/s12939-018-0753-9)
Supplement: Supplementary file 1 — (1) Interview guide for family members, and (2) Interview guide for staff members. (DOCX 103 kb) [file 12939_2018_753_MOESM1_ESM.docx]

*Additional file 1 shows: (1) Interview guide for family members, and (2) Interview guide for staff members.*

**(1)**

**Interview guide. Family member questions.**

Thank you for agreeing to speak to me today. Confidentiality and interview explained.

1. Have you always lived in *[named city]?*
   1. What is your opinion of the neighborhood and community?
2. How many children do you have?
   1. How old are they?
3. What do you do when your child/children has/have emergency health concerns?
   - 1. What do you do when your child/children have ongoing/chronic health concerns?
   1. Does gaining help or care differ depending on the age of your children?
      1. If yes, how?
4. Have you ever needed to see a doctor in an emergency?
   1. If yes, what did you do? / Can you tell me what happened?
   2. What do you normally do when you need to see a doctor? (Non-emergency)
   3. Have you ever seen a family doctor?
      1. Are you able to make appointments when you like with a family doctor?
5. What is your experience of accessing healthcare for either of these problems?
   1. Any positive or negative experiences?
6. Do you or your family have health insurance?
   1. If no, why are you not able to have health insurance?
   2. If yes, do you know what healthcare you can get with your insurance?
      1. If no, why are you unsure?
         1. E.g. Education, awareness
7. Have you ever found it difficult to see a doctor / and gain care?
   1. If no, can you describe to me why you don’t have difficulties seeing a doctor for help?
   2. If yes, why was it difficult?
      1. What was the problem? Were there other problems?
         1. Medicines, etc.

E.G.

- - - 1. Structural: transport availability or cost, distance to travel, accessibility or availability of the service required, lack of resources within hospital, waiting times for services, language barrier, lack of interpretation services.
      2. Education: not knowing what entitled to, not knowing where to go or how to get there, not understanding how medical care works or which service to access.
      3. Economic: cost of travel, cost of healthcare, cost of medicine, lack of health insurance, prioritisation.
  1. (If participant has never been) Why have you never been to see a doctor?
  2. Do these problems stop you from visiting or seeking help in the first place?
     1. Any problem in particular?

1. Are there any other services or people you access for care?
   - 1. E.g. Dentist, optician, pharmacist
   1. Is there anyone or anything else that helps you with your health?
   2. Do you ever go to the pharmacist?
      1. Do you go for health problems? Or to buy toiletries?
      2. Have you had any difficulties with this?
   3. Do you ever see a nurse when you are unwell?
      1. Did you have any difficulties with this?
   4. Have you ever seen a physiotherapist?
   5. Have you ever seen a councilor?
   6. What makes it difficult to visit or access these services or people?

*Refer back to what they have said.*

1. Do these problems you have mentioned, on accessing health care effect or influence you or your child/children’s overall health and well being?
   1. How do they affect you or your child?
   2. How does that make you feel?
   3. Does it create any emotional problems?
2. Are there any other problems you haven’t mentioned, that you face?
   1. If yes, what other problems?
3. Are there things you can do, or are aware you could do, to try and resolve any of these difficulties?
   1. How do think you could get around those problems?
   2. What could you do?
   3. Have you tried any of these suggestions?
      1. If yes, what was the outcome of you trying this?
4. Is there anyone that can help you with the problems you face accessing health care?
   1. Do you ever ask for help?
      1. Who do you ask?
      2. What could they do or do they do to help?
   2. E.G. help with resources, support, and education.
5. Generally, what do you think about the healthcare services within Romania?
   1. What are the positive and negative aspects of it?
   2. Has the ability to access care changed over time? Has it always been the same, or was it different in the past?
6. Is there anything else you’d like to add or tell me?

___________________________________________________________________________

**(2)**

**Interview guide. Staff questions.**

Thank you for agreeing to speak to me today.

Confidentiality and interview explained

1. How long have you worked at the centre?
   1. Can you describe your role within the centre?
2. How long have you lived in *[named city]?*
   1. Where did you used to live?
3. Within your job, how much contact do you have with the families?
4. Generally, what do you think about the healthcare services within Romania?
   1. Positive, negatives
   2. Has the ability to access care changed over time? Has it always been the same or has it been different in the past?

1. In general, in Romania, how do you get an appointment with a family doctor?
   1. How do you get an appointment with a hospital doctor, or get seen at a hospital for an emergency?
   2. Are there any other differences?
2. Are you aware of any challenges or problems that families face when accessing either of these health care services?
   1. If yes, what are the problems faced?
      1. Any problems with accessing doctors or other health care professionals? For example, nurses, pharmacists, councillors
      2. Any issues with accessing certain services? E.g. GP, hospital, council services, pharmacy’s
      3. E.G.
         1. **Structural:**

Transport availability or cost, distance to travel, accessibility or availability of the service required, lack of resources within hospital, waiting times for services, language barrier, lack of interpretation services.

- - - 1. **Educational:**

Not knowing what entitled to, not knowing where to go or how to get there, not understanding how medical care works or which service to access with relation to health insurance.

- - - 1. **Economic:**

Cost of travel, cost of healthcare, cost of medicine, lack of health insurance, priorities (food over health)

- 1. Have you had any experiences directly accessing care with the children?
  2. Do families ever talk to you about their difficulties or come to you or the centre for help with health care?

1. Are you aware of anything else / anything that stops families accessing the care they need?
2. What impact do these barriers have on both the parents/guardians and child’s overall health and well being?
   1. Physically and mentally
   2. Do you see or know of any psychological problems in the community?
3. Have you been involved in helping families with accessing healthcare?
   1. Can you describe some of these experiences?
   2. Are there any trends in health problems that you have noticed?
      1. Children or parents
      2. E.g. emergency, non-emergency
4. Do you think this is a problem for other families in Romania? Not just those in this community?
5. Do you, personally, ever face any problems with accessing health care?
6. What steps, if any, does the centre take to support child health and well-being?
   1. E.G. Do you help with check-ups? E.g. dentist or doctor
   2. What happens if a child needs emergency health care when at the centre?
   3. Has the centre ever had a nurse available/service?
7. How do you think these problems that you have mentioned could be resolved?
   1. *Ask about specific problems highlighted.*
   2. Is there anything the centre does or could do to help these families?
8. Is there anything else you’d like to add or tell me?
